# Social network analysis and whole genome sequencing in a cohort study to investigate TB transmission in an educational setting

**DOI:** 10.1186/s12879-019-3734-8

**Published:** 2019-02-13

**Authors:** Simon Packer, Claire Green, Ellen Brooks-Pollock, Katerina Chaintarli, Sarah Harrison, Charles R. Beck

**Affiliations:** 1 Field Epidemiology Service, Public Health England, Bristol, UK; 2 Heart and Lung Unit, Torbay and South Devon NHS Foundation Trust, Torbay, UK; 3 NIHR Health Protection Research Unit in Evaluation of Interventions at the University of Bristol, Bristol, UK; 4 South West Centre, Public Health England, Totnes, UK; 5 School of Social and Community Medicine, University of Bristol, Bristol, UK

**Keywords:** Tuberculosis, Outbreak, Screening, Whole genome sequencing, Social network analysis

## Abstract

**Background:**

TB outbreaks in educational institutions can result in significant transmission and pose a considerable threat to TB control. Investigation using traditional microbiological and epidemiological tools can lead to imprecise screening strategies due to difficulties characterising complex transmission networks. Application of whole genome sequencing (WGS) and social network analysis can provide additional information that may facilitate rapid directed public health action. We report the utility of these methods in combination with traditional approaches for the first time to investigate a TB outbreak in an educational setting.

**Methods:**

Latent tuberculosis infection (LTBI) cases were screenees with a positive T-SPOT®.*TB* test. Active TB cases were defined through laboratory confirmation of *M. tuberculosis* on culture or through clinical or radiological findings consistent with infection. Epidemiological data were collected from institutional records and screenees. Samples were cultured and analysed using traditional *M. tuberculosis* typing and WGS. We undertook multivariable multinomial regression and social network analysis to identify exposures associated with case status and risk communities.

**Results:**

We identified 189 LTBI cases (13.7% positivity rate) and nine active TB cases from 1377 persons screened. The LTBI positivity rate was 39.1% (99/253) among persons who shared a course with an infectious case (odds ratio 7.3, 95% confidence interval [CI] 5.2 to 10.3). The community structure analysis divided the students into five communities based on connectivity, as opposed to the 11 shared courses. Social network analysis identified that the community including the suspected index case was at significantly elevated risk of active disease (odds ratio 7.5, 95% CI 1.3 to 44.0) and contained eight persons who were lost to follow-up. Five sputum samples underwent WGS, four had zero single nucleotide polymorphism (SNP) differences and one had a single SNP difference.

**Conclusion:**

This study demonstrates the public health impact an undiagnosed case of active TB disease can have in an educational setting within a low incidence area. Social network analysis and whole genome sequencing provided greater insight to evolution of the transmission network and identification of communities of risk. These tools provide further information over traditional epidemiological and microbiological approaches to direct public health action in this setting.

## Background

Tuberculosis (TB) in low incidence settings is often characterised by localised outbreaks. Educational settings represent an environment where a single case can have a large number of close contacts and result in numerous active and latent cases [1–3]. Previous outbreaks in similar settings have reported latent tuberculosis infection (LTBI) rates of 10–40%. The public health response to these outbreaks varied in the extent of screening undertaken (students with/without educational institution staff and members of the public) and differing degrees of contact with the suspected index case (direct case contact or exposure to the same classroom). Transmission is enhanced by a high level of social mixing, shared teaching, communal spaces and close contact between students [1–7].

The threat posed by TB transmission within educational settings requires prompt and focused action to ensure effective TB control. Current NICE guidelines state that a risk assessment should be carried out for sputum smear positive contacts of those who have shared a course whilst the case was potentially infectious. This should be extended to extracurricular activities based on infectious duration, infectivity, population susceptibility, and proximity of contact [8].

There are several epidemiological and health protection challenges associated with TB outbreaks in educational settings, such as: the prioritisation of screening based on infection risk, characterisation of transmission networks and identification of transmission outside of the educational setting [5, 6]. Traditional epidemiological methods, Mycobacterial interspersed repetitive unit variable number tandem repeat (MIRU-VNTR) microbiological typing and the ‘stone in the pond’ approach to inform contact tracing and screening are useful tools for investigating a TB outbreak [8]. Recent developments have seen social network analysis (SNA) and whole genome sequencing (WGS) used to investigate TB outbreaks within community settings [9–11]. SNA has been shown to improve active case finding by highlighting areas of social aggregation and identifying persons not named during traditional contact tracing [9]. WGS provides an increased resolution of microbiological relatedness between isolates compared to MIRU-VNTR, which when combined with epidemiological data improves the capability to confirm or discount outbreaks, identify super spreaders and focus public health action [12, 13]. Whilst these innovative methods have been used in outbreak investigations previously, to our knowledge they have not yet been applied to an outbreak in an educational setting [9–11].

In early 2015, three cases of sputum smear positive pulmonary TB occurred in the South West of England. Two were microbiologically confirmed as the same Beijing strain by MIRU-VNTR typing; the suspected index case was diagnosed abroad and no longer in full time education in England. Whilst no sputum isolate was available for microbiological typing, this individual had symptoms clinically compatible with active TB disease and strong epidemiological links with two cases through attendance at the same educational institution. One of these cases, screened as a close contact of the index case, was in the same year and had left the educational institution the previous summer. This individual had not posed a risk of infection within the educational institution. The other case was diagnosed upon admission to hospital. This case was still attending the school and was two academic years below the suspected index case; the only identified epidemiological link was attendance at the same educational institution. An additional case was identified at a second educational establishment; no epidemiological links to the other cases were identified. A multiagency incident control team was established and led by Public Health England. Upon finding a high number and rate of LTBI positivity among close contacts, students and staff members who attended the educational institution were screened for LTBI and active TB disease.

We report use of traditional epidemiological methods in combination with SNA and WGS to investigate the transmission of TB within the educational institution attended by the suspected index case. We consider implications of applying these innovative methods on screening practice and outbreak control in the future.

## Methods

The ‘stone in a pond’ method was used to identify persons for screening. Close contacts were screened in accordance with NICE guidelines [8]; we subsequently extended screening to students and staff who shared a course with either infectious case. After identifying a high LTBI positivity rate we offered screening to all persons who had studied or worked at the institution. When an additional active case was identified their close contacts were screened as per NICE guidelines [8]. All individuals provided consent prior to screening.

Epidemiological data were collected on all students and staff attending the educational institution during academic years 2013/14 and 2014/15 (the identity of specific year groups have been pseudonymised and we refer to these as year groups A and B). These data included information on courses attended extracted from a database held by the educational institution; data on exposures (ethnic origin, previous contact with a case, travel abroad), Bacillus Calmette-Guérin [BCG], symptoms and socio-demographic characteristics (age, sex and postcode of residence) were collected using a bespoke questionnaire.

We categorised persons screened as low or high risk of LTBI depending on whether they shared a course with one of the two infectious cases. These persons were the only infectious cases attending the educational institution. The remaining Beijing strain case without epidemiological links was defined as a separate index case within their separate educational setting and a distinct screening intervention undertaken.

Screening for TB (active or latent) was undertaken as per NICE guidelines and we used the interferon-gamma release assay T-SPOT®.*TB* test. LTBI positive (defined as positive on T-SPOT®.*TB* test) persons were clinically assessed by the local TB services. LTBI cases were defined as those with a positive T-SPOT®.*TB* test and were asymptomatic and had normal chest radiography. Active cases were defined through laboratory confirmation of *M. tuberculosis* on culture or through a pathologic, radiologic or therapeutic response consistent with active infection. For persons clinically assessed for active TB, sputum samples were cultured and analysed using MIRU-VNTR typing and WGS [14, 15]. Since WGS was not available routinely at the time, isolates were prioritised to enhance inference of the putative TB transmission network and included those obtained from two sputum smear positive cases identified early in the outbreak, the case with epidemiological links to a second educational establishment and two further cases identified through mass screening.

WGS, bioinformatics, and phylogenetic analysis were carried as described previously [15–17]. Briefly, liquid cultures in mycobacterial growth indicator tubes (MGIT tubes; Becton Dickson) were prepared for WGS as described in Pankhurst et al. [18]. These were sequenced using a semi-automated bioinformatics pipeline with species identification using Mykrobe v0.3 [16]. Isolates were mapped onto a library of mycobacterial reference genomes for resistance predictions mapped to the *Mycobacterium tuberculosis* complex reference strain H37Rv by use of previously validated mutation catalogue [15].

Data were merged using personal identifiers (name and date of birth), cleaned and checked for within and cross-consistency errors using pre-defined rules. Descriptive statistics were calculated to describe the population screened and stratified by risk group, student/staff status and LTBI positivity. Missing data related to exposure variables were examined by LTBI status and subsequently excluded from the analysis. Multinomial regression was used post hoc to investigate the association between exposures of interest and three outcome measures of screened and not infected, LTBI positive and active disease. This used a forward additive process based on changes in model and variable significance. *P*-values were calculated using a two-tailed z test.

A post hoc social network analysis was conducted using the igraph package in R. A weighted network was constructed using course lists for the 2 year groups with smear positive cases (year A and year B). The weight of the link between two individuals was defined by the number of shared courses, with no link indicating no shared course. We determined out-degree for each individual as the number of individuals with a shared course and the weighted out-degree as the total number of shared courses. Each individual was assigned to a community (a group of densely connected individuals) within the network, which was determined using the igraph algorithm that maximised modularity (which in this case was the leading eigenvector algorithm) [19]. Analyses were carried out using Stata version 13.1 (StataCorp LLC, College Station, TX, USA) and R version 3.2.0 (R Foundation for Statistical Computing, Vienna, Austria). Ethical approval was not required as the work was part of an outbreak investigation.

## Results

A total of 1377 out of 1570 persons were screened during two sessions, which represented 87.7% of the persons included in the educational institution’s records. Others were screened by the hospital service and results are not included. Persons who attended screening were younger (median difference in age of 1 year, *p* < 0.001) and more likely to have shared a course with an index case (and be classified as high risk of LTBI) (18.9% vs 11.9%, *p* = 0.017) than persons who did not attend screening. The majority of persons screened were students (83.0%). The proportion of males and females was similar (52.7% vs 47.1%). The majority of persons were born in the UK (92.1%), as were both their parents (94.6%). There were 260 (18.9%) persons who shared a course with an index case and therefore were classified as high risk of LTBI.

We identified 189 persons who screened LTBI positive, giving an overall LTBI positivity of 13.7% (*n* = 1377). This percentage varied by high (38.1%, *n* = 260) and low (8.1%, *n* = 1117) LTBI risk groups. We found no LTBI positive persons in one out of 7 year groups. None of these individuals had attended the institution as a student whilst the first infectious case was present. Fewer than five people tested positive in a separate year group. This was considered to be within the expected background rate for LTBI in this area of England.

Ten cases were diagnosed by local TB services with active TB which included two prior to mass screening, seven during and 1 a year after screening had taken place. All active cases experienced pulmonary disease and three were sputum smear positive. All active cases were students, six were male and six had shared a course with the suspected index case. No common extra-curricular activities were identified between the active cases.

Five samples were successfully sequenced using WGS. The average total number of mapped reads was 4.46 million per sample (range 3.09 to 9.2 million), where on average 98.7% (range 98.26 to 99.18%) of these were mapped to the reference genome achieving an average coverage of 91.8% (range 91.7 to 92.0%). All samples were found to be Beijing strain isolates and sensitive to first line drug therapy. Samples from four cases were found to have zero SNP differences between them and one sample had a single SNP difference separating it from these four cases. All samples were found to have identical MIRU-VNTR profiles. Nationally there were no other isolates at the time of the outbreak related by MIRU-VNTR or WGS.

The odds of persons testing positive for LTBI was highest for those sharing a course with either infectious case (odds ratio [OR] 7.3, 95% confidence interval [CI] 5.2 to 10.3). This varied by infectious case and contact with the suspected index case was associated with the highest odds of LTBI (OR 14.2, 95% CI 9.0 to 22.4). The contacts of the second infectious case were also associated with increased odds of LTBI (OR 4.2, 95% CI 2.7 to 6.5). There was an ordinal trend identified between the number of courses shared with the suspected index case and LTBI positivity, with positivity increasing with a greater number of courses (*p* = 0.005). There were 90 cases of LTBI and two active cases who did not share a course with either of the infectious cases.

**Fig. 1:**
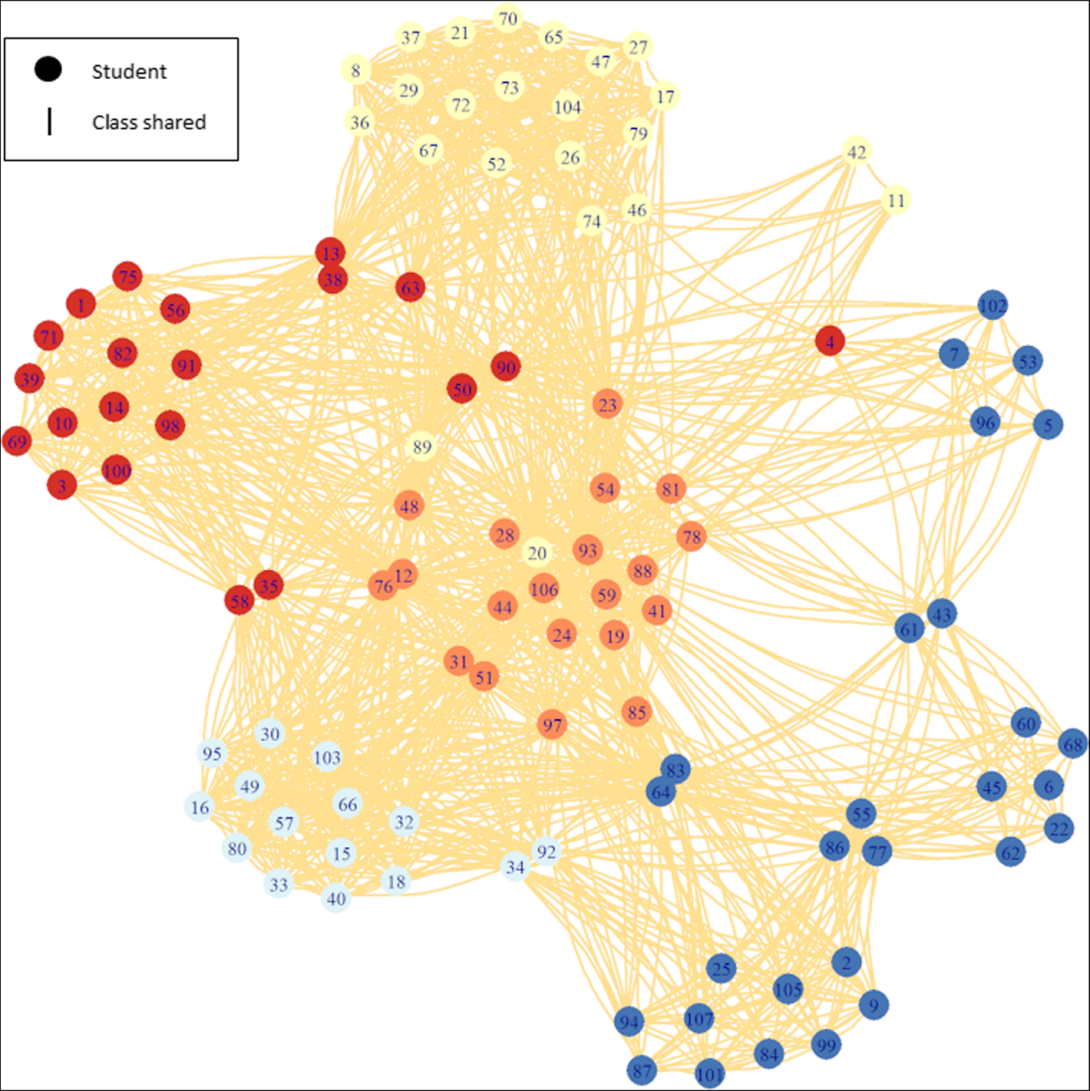
Community analysis of year group B course list data, the division with the largest modularity was used to define community membership. Circles and lines are colour coded to highlight modularity as defined using leading eigenvector algorithm. Branch length is for presentation only

Social network analysis of 2013/2014 year group B course data contained 107 students who shared at least one course with the suspected index case, 17 of whom were not screened. There were seven persons with active disease, 50 with LTBI and 33 who were not infected. The community structure analysis divided the students into five communities based on connectivity, as opposed to the 11 shared courses, with a modularity of 0.43. Each community contained between 15 and 28 students; see Fig. 1.

Two communities were associated with higher rates of LTBI and active disease, which represented 35 students at increased odds compared to the 107 that shared courses with the suspected index case. Importantly, eight of these high risk persons were not screened as they had left the educational institution and were lost to follow up. SNA found that contacts of active disease cases (OR 1.8, 95% CI 1.0 to 3.2), increasing number of courses shared with the suspected index case (OR 2.5, 95% CI 1.4 to 4.8) being part of the same community as the suspected index case (OR 7.5, 95% CI 1.3 to 44.0), the number of courses shared (OR 1.1, 95% CI 1.1 to 1.2) and an increasing number of latent contacts (OR 1.3, 95% CI 1.1 to 1.5) were all associated with increased odds of developing active disease but not testing positive for LTBI; see Table 1.

**Table 1:** Crude odds of infection, LTBI and active disease compares to those who screened negative derived using multinomial social network analysis of year groups A and B course data

Case status	Variable	OR	95% CI	*p*-value
Infected (LTBI or active disease)	Number of active contacts	1.37	1.02–1.84	0.04
	Number of courses with suspected index case	1.69	1.08–2.64	0.02
	Part of suspected index case community	2.23	0.67–7.45	0.19
	Number of courses shared with LTBI cases	1.06	1.01–1.11	0.03
	Number of LTBI contacts	1.10	1.00–1.21	0.06
Active disease	Number of active contacts	1.83	1.04–3.20	0.04
	Number of courses with suspected index case	2.57	1.38–4.79	0.00
	Part of suspected index case community	7.47	1.27–44.00	0.03
	Number of courses shared with LTBI cases	1.13	1.05–1.22	0.00
	Number of LTBI contacts	1.26	1.09–1.45	0.00
LTBI	Number of active contacts	1.25	0.93–1.68	0.15
	Number of courses with suspected index case	1.40	0.91–2.14	0.13
	Part of suspected index case community	1.23	0.37–4.06	0.73
	Number of courses shared with LTBI cases	1.03	0.98–1.09	0.19
	Number of LTBI contacts	1.06	0.96–1.18	0.25
Not screened	Number of active contacts	1.65	1.11–2.45	0.01
	Number of courses with suspected index case	1.08	0.60–1.94	0.81
	Part of suspected index case community	0.75	0.13–4.32	0.74
	Number of courses shared with LTBI cases	1.03	0.96–1.10	0.41
	Number of LTBI contacts	1.11	0.98–1.26	0.09

*LTBI* latent tuberculosis infection, *OR* odds ratio, *CI* confidence interval

The univariate multinomial analysis including all persons screened identified age in years (OR 1.3, 95% CI 1.2 to 1.5), pupil status (OR 1.7, 95% CI 1.0 to 2.7) and male sex (OR 1.6, 95% CI 1.2 to 2.2) as associated with LTBI. Developing active disease was associated with age (OR 1.9, 95% CI 1.3 to 2.8). The multivariable analysis found student status (adjusted odds ratio [aOR] 9.4, 95% CI 1.9 to 45.7) and presence of BCG (aOR 1.8, 95% CI 1.1 to 3.0) was positively associated with the odds of acquiring latent disease. Active disease remained positively associated with age in years (aOR 2.0, 95% CI 1.2 to 3.5); see Table 2.

**Table 2:** Crude and adjusted odds of infection, LTBI and active disease compared to those who screened negative derived using multivariate multinomial analysis of the educational institution

Case status	Variable	OR	95% CI	*p*-value	aOR	95% CI	*p*-value
Infected (LTBI or active disease)	Age (years)	1.35	1.23–1.48	0.00	1.04	1.00–1.08	0.06
	Pupil	1.77	1.11–2.83	0.02	10.69	2.14–53.3	0.00
	Male	1.62	1.19–2.19	0.00	1.26	0.89–1.77	0.19
	BCG	1.03	0.69–1.54	0.90	1.76	1.07–2.89	0.03
	Sibling	1.13	0.88–1.46	0.34	1.30	0.99–1.72	0.06
	Live abroad	1.34	0.79–2.29	0.28	1.44	0.77–2.68	0.25
LTBI	Age (years)	1.32	1.2–1.45	0.00	1.04	0.99–1.08	0.09
	Pupil	1.66	1.04–2.66	0.03	9.36	1.91–45.79	0.01
	Male	1.59	1.17–2.17	0.00	1.25	0.88–1.77	0.21
	BCG	1.07	0.71–1.61	0.74	1.82	1.11–3.01	0.02
	Sibling	1.16	0.89–1.5	0.28	1.30	0.98–1.72	0.07
	Live abroad	1.40	0.82–2.39	0.22	1.48	0.79–2.75	0.22
Active	Age (years)	1.91	1.29–2.83	0.00	2.01	1.16–3.48	0.01
	Pupil^a^	–	–	–	–	–	–
	Male	2.11	0.61–7.24	0.24	1.68	0.27–10.28	0.58
	BCG	0.00	0.00–0.00	0.00	0.00	0.00–0.00	0.00
	Sibling	0.76	0.23–2.49	0.65	1.84	0.48–7.06	0.38
	Live abroad	0.00	0.00–0.00	0.00	0.00	0.00–0.00	0.00

*LTBI* latent tuberculosis infection, *BCG* Bacillus Calmette-Guérin, *OR* odds ratio, *aOR* adjusted odds ratio, *CI* confidence interval

^a^ all the active cases were pupils

## Discussion

We identified substantial transmission within an educational institution located in an area with a low background rate of TB in the community. We hypothesise this was predominately due to a single infectious case with smear positive disease and a high bacterial load. This is illustrated by the higher odds of LTBI associated with sharing a course with the suspected index case compared to the second infectious case, and how specific communities were associated with substantially greater odds of LTBI. The data indicates that the second case propagated the outbreak but to a lesser extent, however, we are unable to discount that the suspected index case was the source of all transmission events. The presence of a single SNP differences between isolates which underwent WGS suggests contemporaneous transmission to or between cases. It is important to note that none of the other active cases were infectious whilst attending the educational institution. Close contact screening was undertaken as per NICE guidelines; these persons were not included in the analysis [8].

The multinomial analysis found students to have the highest odds of LTBI acquisition and this is likely due to the intensive social mixing of students with the two infectious cases compared to staff who worked at the educational institution. Educational institutions are semi-closed settings where students have close and prolonged contact with each other, there is a high degree of social mixing and classrooms can be poorly ventilated making them an ideal environment for transmission [5, 7]. In this outbreak, we observed that transmission can extend beyond direct contact in the classroom due to LTBI cases in persons who did not share a course with either infectious case. This emphasises the importance of shared airspace in classrooms and places of social aggregation within the educational institution [6]. Consequently, the public health response to TB outbreaks in educational settings should look at shared airspace and opportunities for social mixing in addition to direct course sharing. This is in addition to current NICE guidelines [8].

Following this outbreak additional cases with the same MIRU-VNTR profile that are not students or members of staff at the educational institution have been identified. Three had epidemiological links to the educational institution, leaving three cases with no epidemiological links but with links to the same geographical area. This shows that the transmission network has extended into the wider community where ongoing routine surveillance is important to ensure case ascertainment and local TB control.

Delay in diagnosis has frequently been cited as an important factor in previous outbreaks and played a role in this outbreak. In low incidence areas, clinical exposure and awareness surrounding the signs and symptoms of TB contributes to delays [1, 2, 5, 6]. Awareness around TB in both educational staff and primary care doctors is important as delays can result in prolonged opportunities for transmission and a greater public health impact on the population at risk.

Health protection measures were implemented alongside the epidemiological investigation being undertaken. This outbreak was originally investigated using traditional epidemiological and microbiological methods which were later supplemented by WGS and SNA. Our study demonstrates that WGS provides added inference regarding temporality of TB transmission over and above MIRU-VNTR reference typing, which supported the multiagency incident control team to describe key aspects of the putative TB transmission network and focus public health action accordingly. WGS is currently being introduced as a routinely available service for TB isolates across England and provides numerous advantages compared to MIRU-VNTR reference typing. In addition to enhanced strain discrimination and relatedness, laboratory turnaround times are substantially reduced and inferences regarding transmission between cases can guide control measures in real-time [20]. The discriminatory power of WGS precisely defines clusters of genetically related isolates and allows for highly sensitive and specific case definitions [9, 12, 20]. This decreases misclassification bias in epidemiological studies, improves resource allocation and public health risk assessment. The ability to estimate time since transmission and the corresponding directionality provides information to support contact tracing, understand transmission networks and identify at-risk persons. MIRU-VNTR typing cannot accurately capture this genomic diversity and is associated with lower precision and misclassification [9]. The advantages of WGS been demonstrated in previous studies and outbreak investigations and were similarly observed within our investigation [9, 10, 12].

The SNA analysis allowed visualisation of course sharing in greater detail and demonstrated the nature of transmission in this setting. We found that certain courses or combinations of courses (communities) had a greater number of LTBI positive students, suggesting enhanced transmission within key course sharing groups. We found that SNA can add greater precision and accuracy to traditional ‘stone in the pond’ contact tracing in an outbreak setting [9]. These findings combined with the factors identified in the multinomial analysis provided a high level of detail and these methods can be used to prioritise screening interventions in future outbreaks. For example, we identified eight persons within the high risk community who were not screened (as they had left the educational institution and were lost to follow up). Traditional contact tracing methods identified these cases but prioritised them alongside a large number of lower risk cases. Social networks can be produced based upon course sharing, attendance of extracurricular activities in relation to the index case, or any other unifying activities or shared exposures. Further iterations of this network can be produced as screening results are obtained and can be used to both refine and validate the network as well as direct future screening activities.

Strengths of this study include its large and comprehensive sample. There was a high response and a large number of persons were screened. This limited sampling bias and provided sufficient data for analysis. The innovative methods used, WGS and SNA, provided further insight to the outbreak and potential areas to guide public health intervention over traditional methods. We made use of multiple sources of data and combined them to give insights into TB epidemiology in an educational setting; furthermore it confirmed that the action the incident control team had taken was correct.

The nature of an outbreak provides strong ecological validity however the challenges posed by precision of data collection and resulting issues with completeness will undoubtedly affect the findings of this study. The limitations were that the SNA was done following the initial outbreak investigation and therefore could not be used to prioritise cases for screening. Prior administration of BCG was self-reported and liable to recall bias which could affect the estimates in either direction and likely explains the incongruous association found in our data. In particular, we understand that some people believed that it was part of routine immunisation and responded affirmatively if their children were otherwise fully immunised. The investigation occurred over two different years which meant that people left and enrolled in the educational institution. This complicated contact tracing and altered the data collected and the exposure status of these groups.

This manuscript and others have shown that introduction of an infectious student to an educational setting can result in a large number of both latent and active TB cases [1–7]. This puts a logistical and financial strain on local clinical and public health services, and threatens TB control efforts. The suspected index case in this outbreak was an international student from a high burden country. Those entering the UK since May 2012 undergo active TB screening as part of UK entry regulations and whilst new migrant LTBI screening was introduced in 2016, funding has not been made routinely available to low incidence areas of the country [8, 9]. A large number of boarding schools and universities are located in rural low incidence areas and therefore students, including within our study, will not be eligible for LTBI screening whereas they were previously [21]. A study in Canada showed that when LTBI therapy was considered as a marginal cost (included as part of an existing screening programme) it was highly cost-effective [21]. We recommend health economic studies are undertaken in England, modelling the impact of screening all persons applying for a student visa for LTBI as part of their routine pre-entry active TB screening assessment. Finally, we recommend that SNA and WGS are considered when investigating TB outbreaks in educational settings, and potentially other closed and semi-closed settings.

## Conclusion

This study demonstrates the public health impact an undiagnosed case of active TB disease can have in an educational setting within a low incidence area. Outbreaks of this kind put considerable strain on TB services and often result in a large number of active and latent cases. In this investigation the combination of genomic and epidemiological data provided strong evidence upon which strong inferences could be made. The innovative methods used were beneficial and should be considered for use by incident control teams when investigating future outbreaks in similar settings. This study supports the need for epidemiological data in addition to WGS to confirm an outbreak and describe the directionality of transmission [12].

## Abbreviations

aOR: Adjusted odds ratio
BCG: Bacillus Calmette-Guérin
LTBI: Latent tuberculosis infection
MIRU-VNTR: Mycobacterial interspersed repetitive unit variable number tandem repeat
NIHR HPRU: National Institute for Health Research Health Protection Research Unit
OR: Odds ratio
PHE: Public Health England
SNA: Social network analysis
TB: Tuberculosis
WGS: Whole genome sequencing
